# Calcium Carbonate Cement: A Carbon Capture, Utilization, and Storage (CCUS) Technique

**DOI:** 10.3390/ma14112709

**Published:** 2021-05-21

**Authors:** Craig W. Hargis, Irvin A. Chen, Martin Devenney, Miguel J. Fernandez, Ryan J. Gilliam, Ryan P. Thatcher

**Affiliations:** 1 Fortera Corporation, 251 E. Hacienda Ave, Suite B, Campbell, CA 95008, USA; rgilliam@forterausa.com (R.J.G.); rthatcher@forterausa.com (R.P.T.); 2 Calera Corporation, Los Gatos, CA 95032, USA; irvinachen@gmail.com (I.A.C.); mdevenney@gmail.com (M.D.); mfernandez@calera.com (M.J.F.)

**Keywords:** cement, calcium carbonate, vaterite, carbon capture, industrial ecology

## Abstract

A novel calcium carbonate cement system that mimics the naturally occurring mineralization process of carbon dioxide to biogenic or geologic calcium carbonate deposits was developed utilizing carbon dioxide-containing flue gas and high-calcium industrial solid waste as raw materials. The calcium carbonate cement reaction is based on the polymorphic transformation from metastable vaterite to aragonite and can achieve >40 MPa compressive strength. Due to its unique properties, the calcium carbonate cement is well suited for building materials applications with controlled factory manufacturing processes that can take advantage of its rapid curing at elevated temperatures and lower density for competitive advantages. Examples of suitable applications are lightweight fiber cement board and aerated concrete. The new cement system described is an environmentally sustainable alternative cement that can be carbon negative, meaning more carbon dioxide is captured during its manufacture than is emitted.

## 1. Introduction

Anthropogenic carbon dioxide (CO_2_) is contributing to the rising atmospheric CO_2_ concentration, which is widely considered to be a leading contributing factor for the greenhouse effect, ocean acidification, and global climate change [1]. While efforts are being made to reduce CO_2_ emissions, global anthropogenic CO_2_ emissions are projected to continually increase over the coming decades, mainly driven by the growth of emerging countries [1,2,3,4,5,6,7,8,9]. Carbonate rocks are estimated to contain approximately 39,000,000 Gt of terrestrial CO_2_ and provide a potential pathway for stable, long-term storage of CO_2_ resulting from human activities [10]. Furthermore, carbonate rocks, such as limestone and marble, both mainly composed of calcium carbonate (CaCO_3_), have long been widely utilized by mankind as construction materials, which offer a potential solution to upcycle the anthropogenic CO_2_ into value added materials [11].

CaCO_3_, one of the most abundant carbonate species in nature, has three anhydrous crystalline polymorphs: vaterite, aragonite, and calcite, with vaterite being the least and calcite being the most thermodynamically stable polymorph [12]. Calcite is the most abundant polymorph and is seen in geological formations, such as limestone, chalk, and marble of either biological or geological origin. Aragonite is less common, but abundant as oolitic sand in the Bahamas, while vaterite is observed much less often due to its instability in ambient conditions. However, in nature, many organisms, e.g., coral, first produce metastable amorphous CaCO_3_ as a precursor and control its transformation to aragonite or calcite to develop their rigid skeletons [13].

Inspired by the biological use of CaCO_3_, Fontaine et al. [14] and Combes et al. [15,16] successfully developed a CaCO_3_-based medical cement through an aqueous precipitation process using calcium chloride (CaCl_2_) and sodium carbonate (Na_2_CO_3_) as feedstocks. The cementing reaction is based on the polymorphic transformation from metastable amorphous CaCO_3_ and vaterite to aragonite or calcite through a dissolution–reprecipitation process in an aqueous medium. However, limited production scale and mechanical properties (3–13 MPa compressive strength) have restricted larger scale applications in the construction industry. Nevertheless, the results obtained by Combes et al. provide promising insights for the development of a nature inspired, synthetic CaCO_3_ cement system at scale from anthropogenic CO_2_ with properties suitable for various building materials applications.

Industrially, three feedstocks are required to produce CaCO_3_ cement: CO_2_, calcium, and alkalinity. CO_2_ can originate from CO_2_-containing industrial flue gas, e.g., thermal power plants, chemical plants, and cement kilns. Calcium and alkalinity can come from industrial waste streams that contain both calcium and alkalinity in the form of calcium oxide (CaO), calcium hydroxide (Ca(OH)_2_), and calcium silicates. Examples of calcium and alkalinity-rich industrial waste streams include: carbide lime sludge, an impure Ca(OH)_2_ by-product of producing acetylene gas (C_2_H_2_) from coal and limestone, slag, a Ca/Mg-rich aluminosilicate glass by-product of iron and steel production, and cement kiln dust, an impure CaCO_3_/CaO by-product of Portland cement manufacturing that is often recycled in the cement kiln feed [17,18]. An acid can be utilized to extract the calcium from the waste feedstock and bring it into solution. Ammonium salts, e.g., ammonium chloride (NH_4_Cl), ammonium sulfate ((NH_4_)_2_SO_4_), and ammonium nitrate (NH_4_NO_3_) have been shown to be effective acids in the production of vaterite from CO_2_ because they can bring the calcium into solution (Equation (1)), absorb CO_2_ from a gas stream (Equation (2)), and then be regenerated during the formation of CaCO_3_ (Equation (3)) [17,19,20].

2NH_4_Cl_(aq)_ + CaO_(s)_ + H_2_O_(l)_ → 2NH_4_OH_(aq)_ + CaCl_2(aq)_ (1)

2NH_4_OH_(aq)_ + CaCl_2(aq)_ + CO_2(g)_ → (NH_4_)_2_CO_3(aq)_ + CaCl_2(aq)_ + H_2_O_(l)_ (2)

(NH_4_)_2_CO_3(aq)_ + CaCl_2(aq)_ → 2NH_4_Cl_(aq)_ + CaCO_3(s)_ (3)

To promote the formation of metastable forms of CaCO_3_ over calcite and aragonite, precipitation is performed at high reactant concentrations, needed to achieve high supersaturation and kinetic stabilization [21]. The concentration of carbonate ions is predominantly governed by (1) the rate of transfer of CO_2_ across the gas–liquid interface, (2) the rate at which CO_2_ converts into carbonate, and (3) the carbonate–bicarbonate equilibrium. The rate of mass transfer can be increased by increasing the flow rate of gas or by increasing the gas–liquid interface, for example by high shear mixing. Subsequent conversion of CO_2_ into carbonate can occur through the reaction with water (H_2_O) or hydroxyl ions (OH^−^), but because the latter reaction is 10^7^ times faster, higher rates can be achieved by performing the reaction at higher pH. Higher pH also favors higher equilibrium concentrations of carbonate over bicarbonate. Except for high gas flow rates, these strategies also improve the CO_2_ absorption efficiency.

Herein, a novel CaCO_3_ cement system that mimics the naturally occurring mineralization process of CO_2_ to biogenic or geologic CaCO_3_ deposits is described [17,22]. The CaCO_3_ cement is produced by capturing CO_2_-containing industrial flue gas and utilizing a calcium and alkalinity-rich industrial waste stream [18]. The result is a cement with a significantly lower CO_2_ and energy footprint compared to traditional inorganic binders, such as Portland cement. Furthermore, the CaCO_3_ cement offers processing and performance advantages in a variety of building materials applications over traditional inorganic binders.

## 2. Materials and Methods

### 2.1. Raw Materials

The carbide lime sludge (Gilmour & Company, Inc., Vancouver, WA, USA) contained 25 wt% solids and had silica, alumina, and mixed oxidation state sulfur impurities, e.g., S^2−^ and SO_4_^2−^, see Table 1. The CO_2_-containing flue gas used was generated from a boiler simulator (GE Energy and Environmental Research Corporation, Irvine, CA, USA) burning propane. NH_4_Cl (Zaclon, LLC, Cleveland, OH, USA) was used for solubilizing Ca(OH)_2_.

### 2.2. Aqueous Mineralization Process

**Figure 1:**
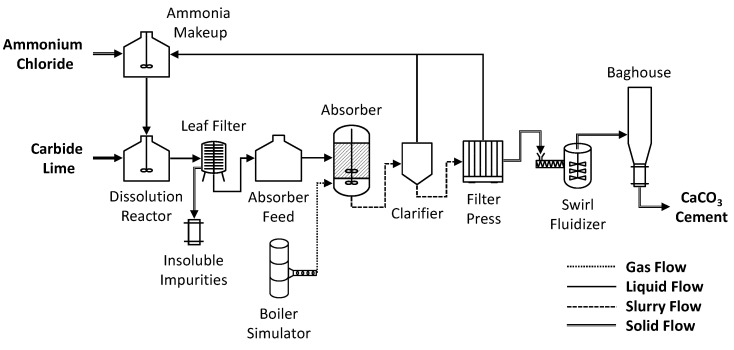
Block flow diagram of the CaCO_3_ cement manufacturing process.

**Figure 2:**
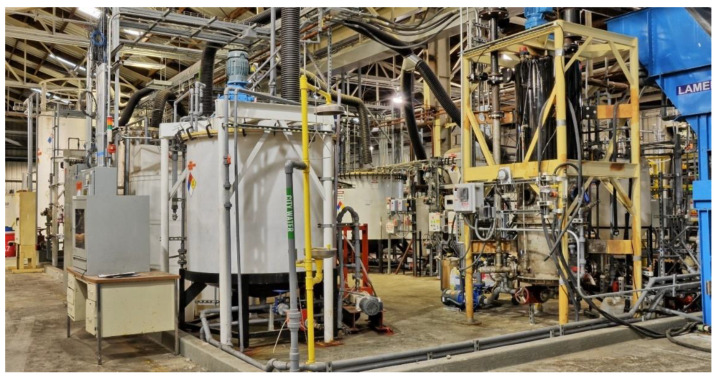
CaCO_3_ cement pilot plant.

To produce CaCO_3_ cement, the carbide lime sludge is first solubilized with aqueous NH_4_Cl then passed through a leaf filter to remove insoluble impurities resulting in an aqueous solution of CaCl_2_ and ammonia (NH_3_), see Equation (1). NH_3_ dissolved in water is in equilibrium with ammonium hydroxide (NH_4_OH). CO_2_-containing flue gas (11 vol%) is then contacted with the solution in a three-phase continuous-stirred tank reactor (CSTR) controlled at below 40 °C (Equation (2)), resulting in the precipitation of CaCO_3_ cement (Equation (3)). The simulated flue gas was blown from the boiler simulator to the absorber via a 20 cm steel pipe using a 600 cfm roots style blower. Unlike some other carbon capture technologies, the CO_2_ does not need to be concentrated or compressed to high pressures for storage, which is energy intensive. The CaCO_3_ cement is then mechanically dewatered by a filter press (Outotec Oyj, Helsinki, Finland) and thermally dried in a swirl fluidizer (GEA Processing Engineering, Warrington, UK) to a free-flowing powder. The NH_4_Cl solution is recovered during dewatering and is recycled for the solubilization of additional carbide lime sludge. Insoluble impurities accounted for 7% of the dry mass of the carbide lime sludge and were composed of 70% mixed oxides and 30% carbon, see Table 1. The insoluble impurities were treated as waste at the pilot plant, but, depending on the composition, could be used in industrial ecology applications, e.g., raw materials for Portland cement production or as supplementary cementitious materials [23]. Figure 1 shows a process block flow diagram of the CaCO_3_ cement manufacturing process. The CaCO_3_ cement pilot plant is shown in Figure 2.

### 2.3. Analysis and Testing

#### 2.3.1. Laser Particle Size Distribution

A Horiba LA-950V2 Laser Scattering Particle Size Distribution Analyzer (two light sources: 650 nm wavelength laser and 405 nm wavelength LED) was used to study the particle size distribution of the CaCO_3_ cement. Measurements were carried out with test samples dispersed in isopropyl alcohol using refractive indexes of 1.378 for isopropyl alcohol and 1.58 for CaCO_3_.

#### 2.3.2. X-ray Florescence

An ARL QUANT’X Energy Dispersive X-ray Fluorescence Spectrometer (Thermo Fisher Scientific, Waltham, MA, USA) was used to evaluate the chemical composition of the oven-dried raw materials and the CaCO_3_ cement. The loss on ignition (LOI) was determined by measuring the mass loss after heating the samples in an electric muffle furnace (Thermo Fisher Scientific, Waltham, MA, USA) at 950 °C for 60 min.

#### 2.3.3. X-ray Diffraction

A X’Pert Pro X-ray Diffractometer (Panalytical, Almelo, Netherlands) was used to determine the phase composition of the CaCO_3_ cement and the transformed cement pastes. The instrument was operated at 40 kV and 30 mA (Cu K_α1_, λ = 1.5406 Å) using a scan range of 5–65° 2θ, a step size of 0.02°, and dwell time of 4 s. The resulting spectra were analyzed using Jade 9 software (Materials Data, Inc., Livermore, CA, USA) for qualitative phase composition information and MAUD software for quantitative Rietveld analysis (QXRD) [24].

#### 2.3.4. Scanning Electron Microscopy

A SU-6600 Field Emission Scanning Electron Microscope (SEM) (Hitachi High-Tech, Omuta, Fukuoka, Japan) was used to collect images of the CaCO_3_ cement and the transformed cement paste. The samples were coated with platinum/palladium using a sputter coater (Electron Microscopy Sciences, Hatfield, PA, USA) prior to secondary electron imaging.

#### 2.3.5. Compressive Strength

Compressive strength development of the CaCO_3_ cement was evaluated at 4 h, 10 h, 1 day, 3 days, 7 days, and 14 days. Paste cubes (50 × 50 × 50 mm^3^) with a solution-to-solid ratio (*w/c*) of 0.35 were utilized. The solution contained 0.1 M magnesium and strontium chloride. The pastes were mixed according to ASTM C305 and then cast and compressed according to ASTM C109 [25,26]. The pastes cured inside brass cube molds at the specified temperature (40, 60 or 80 °C) with >95% relative humidity for up to 1 day. For testing between 4 h and 1 day, the paste cubes received no additional curing outside their molds. For 3-, 7-, and 14-day tests, the paste cubes were demolded at 24 h and placed in a bath with the same composition as the mix solution at the specified temperature (40, 60, or 80 °C). The paste cubes were removed from their curing environments at the specified time and dried in a 105 °C oven for 4 h before compressive strength testing with a loading rate of 1 kN/s.

## 3. Results

### 3.1. CaCO_3_ Cement Characteristics

**Figure 3:**
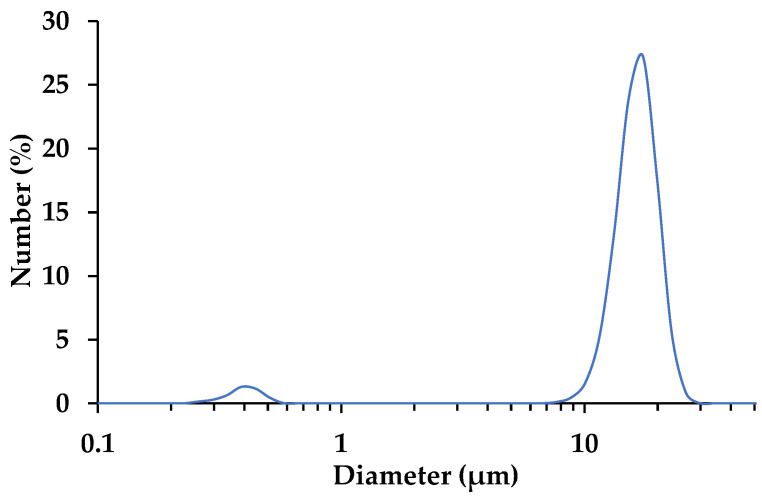
Particle size distribution of the CaCO_3_ cement.

**Figure 4:**
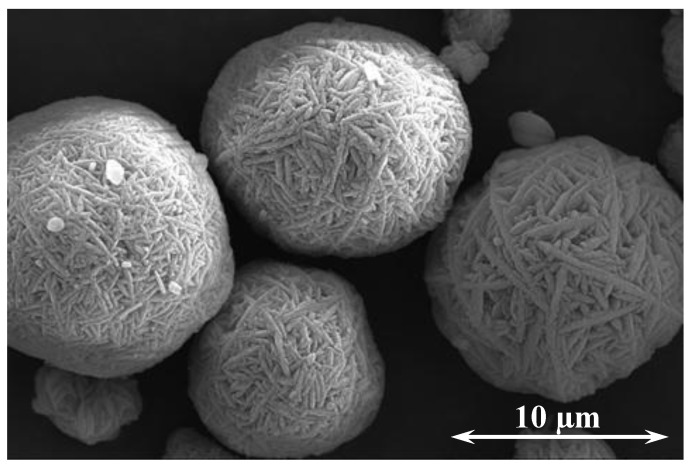
SEM image of the metastable CaCO_3_ cement (vaterite).

The CaCO_3_ cement has a median particle size of 15 with a 4 µm standard deviation. The D_90_ and D_10_ for the CaCO_3_ cement are 19 and 11 µm, respectively. Figure 3 gives the particle size distribution of the cement. Individual crystals and small agglomerates make up less than 4% of the material at a median particle size of 0.4 µm. The primary nucleated crystals agglomerate in the CSTR to minimize their surface energy, forming a yarn-ball like, spherical morphology assembled of several microplates and lenses (Figure 4). Ammonium ions have been shown to promote the growth of vaterite into hexagonal plates and lenses by adsorbing onto the negatively charged (001) plane, which stabilizes the (001) face promoting the hexagonal crystal growth. Together with the oriented attachment of amorphous precursors and/or nanocrystals, the lens shaped single crystals are developed as the crystal grows [27,28,29]. In Figure 4, an individual lens can be observed above the right most vaterite sphere. In diffusion-based experiments, rosette mesostructures developed because the crystal structures were grown on a flat surface; whereas, in this study the mesostructures are spherical because the crystal agglomerates formed in solution.

**Figure 5:**
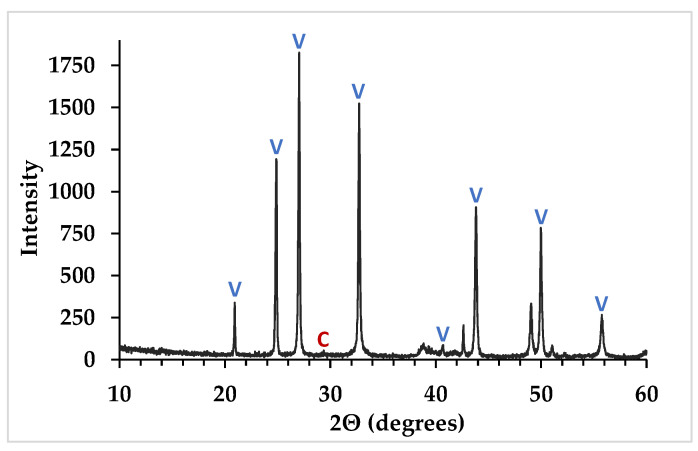
XRD pattern of CaCO_3_ cement (V = vaterite; C = calcite).

Table 2 shows the chemical and mineral composition of the CaCO_3_ cement. The alumina and silica impurities found in the carbide lime sludge are not found in the resulting cement because they are relatively insoluble impurities that were removed during the filtering process (Figure 1); however, some magnesium and sulfate from the carbide lime sludge do make it into the resulting cement. The chloride in the cement comes from the NH_4_Cl solution. The mesostructure of the crystal agglomerates creates porosity and high surface area that traps solution. When the cement is heated and dried, the NH_4_Cl decomposes into hydrogen chloride (HCl) and NH_3_ gas. The HCl and NH_3_ gas are recovered while scrubbing the flue gas. A portion of the HCl gas contacts water on the drying CaCO_3_, forming hydrochloric acid, and is neutralized by the CaCO_3_ cement, leaving a residual amount of CaCl_2_ [30]. The CaCO_3_ cement produced is mainly composed of metastable vaterite (>99 wt%) with a minor amount of calcite (<1 wt%), see Figure 5. Formation of amorphous CaCO_3_ is deliberately avoided as it is difficult to stabilize and process on an industrial scale. Once the CaCO_3_ cement is dried, it is stable under ambient conditions.

### 3.2. Cementitious Reaction

**Figure 6:**
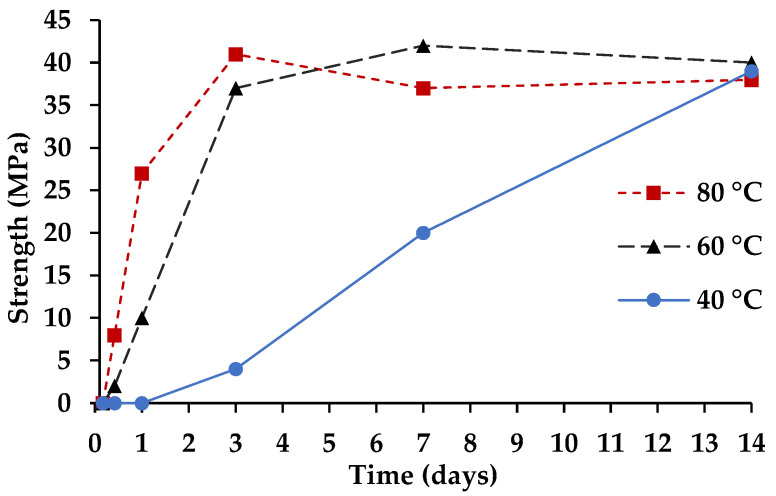
Temperature dependency of CaCO_3_ cement strength development.

The cementing reaction in CaCO_3_ cement is the transformation of vaterite (CaCO_3_) to aragonite (CaCO_3_) or calcite (CaCO_3_). Vaterite dissolves and aragonite and/or calcite precipitates. Since the cementing reaction is an aqueous dissolution-reprecipitation process, curing the CaCO_3_ cement requires high humidity to maintain the transformation of vaterite to aragonite and/or calcite. If the humidity of the curing environment is not controlled, the material will desiccate, and the cementing reaction will cease, similar to Portland cement. Additionally, like Portland cement, the cementing reaction of CaCO_3_ cement can be accelerated by increasing the curing temperature. Figure 6 shows the temperature dependency of the CaCO_3_ cementing reaction and compressive strength development. The ultimate compressive strength of the material is 40 MPa, which is 3–13× higher than the 3–13 MPa compressive strength, previously reported for CaCO_3_ cement developed for medical applications [14,15]. At 40 °C, CaCO_3_ cement paste does not show strength development through 1 day and takes 14 days to achieve the full strength. By elevating the temperature, full strength can be achieved by 7 or 3 days for 60 and 80 °C, respectively. Additionally, at elevated temperature, CaCO_3_ cement pastes have developed enough strength by 4 to 10 h (80 and 60 °C, respectively) to be demolded and handled. Fast early strength is important for manufactured building materials with the need to transfer materials between initial and final curing steps, e.g., from pre-curing in molds at lower temperatures to autoclaving in stacks at elevated temperatures.

During the dissolution of vaterite, aragonite is the preferred precipitate over the most thermodynamically stable calcite, as aragonite exhibits an acicular morphology which can lead to an interconnected microstructure. The formation of aragonite can be controlled through the use of magnesium-based additives (e.g., MgCl_2_, MgSO_4_, Mg(C_2_H_3_O_2_)_2_), which are known to inhibit calcite growth [31,32]. The size and aspect ratio of the acicular aragonite can be controlled by the use of strontium-based additives (e.g., SrCl_2_, Sr(C_2_H_3_O_2_)_2_), which are known to promote aragonite formation [33], resulting in thinner aragonite crystals with higher aspect ratios.

**Figure 7:**
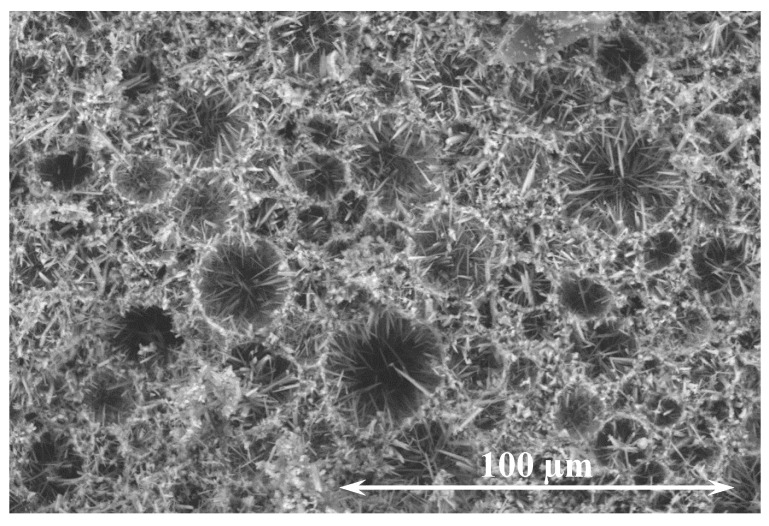
SEM image of the hardened cement paste (predominately aragonite).

The interconnection of the aragonite crystals is controlled through the vaterite particle structure. The spherical vaterite particles have a large (15 µm median size) and heterogeneous structure. At all curing temperatures, aragonite crystals nucleate on the surface of vaterite particles, then grow outward, which results in a dense and interconnected honeycomb microstructure (Figure 7). This phenomenon is consistent to Nielsen et al.’s [34] observation where they hypothesized that the phenomenon resulted from the high mobility and exchange of surface ions with solution. For the CaCO_3_ cement, water is only a medium for the metastable vaterite to dissolve and reprecipitate as aragonite and does not become chemically bonded. All the initial mix water will remain in the system after the cementing reaction and will become porosity after drying. Therefore, the cemented material is inherently more porous and less dense than traditional cements that are based on hydration reactions which chemically bind water. Furthermore, the pore solution of the CaCO_3_ cement paste is close to neutral compared to the alkaline traditional cements.

**Figure 8:**
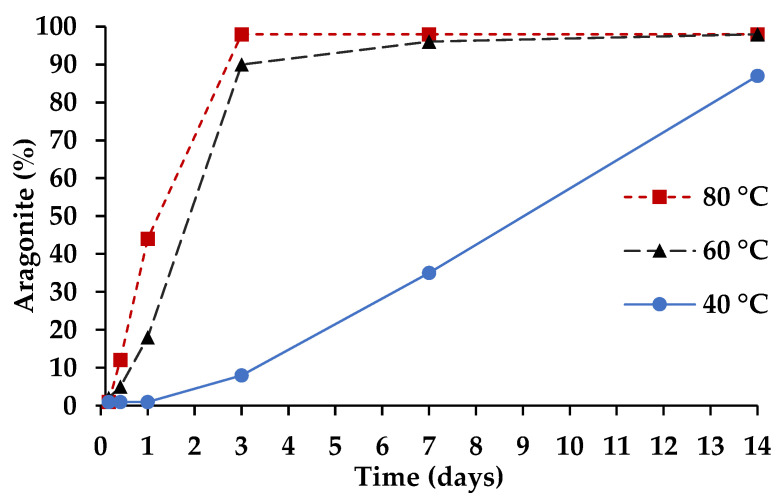
Conversion of CaCO_3_ cement to aragonite as a function of time and temperature (quantified by QXRD).

Figure 8 shows the conversion of the CaCO_3_ cement (vaterite) to aragonite in the hardened cement paste. Comparing Figure 6 to Figure 8 elucidates that the cementing reaction and mechanical property development of the CaCO_3_ cement is entirely dependent on the vaterite to aragonite transformation, which is less complex than traditional cements and can be easily accelerated through temperature without negative effects on performance. For instance, at 80 °C, the CaCO_3_ cement (99% vaterite and 1% calcite) has fully transformed to 98% aragonite and 2% calcite by 3 days of curing; whereas, at 40 °C, the hardened cement paste is composed of 12% vaterite, 87% aragonite and 1% calcite at 14 days of curing.

## 4. Discussion

### 4.1. Raw Materials Availability

Annual global production of carbide lime sludge was estimated at approximately 25 million tons on a dry basis, where >95% is generated in China [23]. Carbide lime sludge typically contains over 90 wt% Ca(OH)_2_ on a dry basis with minor impurities such as carbon, silica, alumina, iron, and sulfur species depending on the composition of coal and limestone. Carbide lime sludge is used in China to replace limestone as a raw material for Portland cement production which can reduce CO_2_ emissions yet increase energy demand depending on the additional energy required to dry the carbide lime [35]. However, there is still a significant amount of carbide lime sludge being landfilled and legacy ponds, which cause major environmental issues due to carbide lime sludge’s high alkalinity (pH 12.4).

Iron and steel slag production is, on the contrary, more evenly distributed worldwide. It is estimated that about 510–664 million tons of iron and steel slag were generated annually in 2019 [36]. Iron and steel slags are composed of about 35–60 wt% CaO with other constituents such as silica, alumina, magnesia, and sulfur species. Except ground granulated blast furnace slag, which is used as a supplementary cementitious material to replace Portland cement in mortar and concrete applications, most iron and steel slags are landfilled or can only be utilized in low-value applications such as road stabilization or asphalt aggregates [37]. Based on both the annual production of waste feedstocks (carbide lime sludge and iron and steel slag) and the CaO extraction efficiency, approximately 100 million tons of CO_2_ could be captured from industrial facilities and approximately 230 million tons of CaCO_3_ cement could be produced.

### 4.2. Building Material Applications

With its unique properties, CaCO_3_ cement is well suited for building materials applications with controlled factory manufacturing processes that are typically made from inorganic binders, such as Portland cement or gypsum. The CaCO_3_ cement can provide processing and performance advantages over Portland cement, e.g., shorter production cycles, lighter weight, and flexibility on fillers and reinforcement elements. Furthermore, CaCO_3_ cement is white, which makes it easy to color and texturize for decorative purposes.

Because of its neutral pore solution, CaCO_3_ cement is not suitable for structural applications that require mild steel reinforcement but is suited for composite systems development with the flexibility to incorporate a wide range of reinforcement elements for superior toughness and flexural properties. The neutral pore solution of the CaCO_3_ cement also makes it amenable for use in environmentally sensitive areas and applications that require biocompatibility.

CaCO_3_ cement’s porosity has inherent advantages and disadvantages. The advantage is the ability to formulate lightweight products. For example, suitable potential applications are lightweight fiber cement board and autoclaved aerated concrete due to their manufacture in controlled environments that utilize elevated curing temperatures to accelerate property development. In other applications, the porosity could be a disadvantage, such as concrete in severe freeze–thaw or physical salt attack environments. The durability of CaCO_3_ cement in a range of situations and applications should be investigated in future research projects.

### 4.3. CaCO_3_ Cement Manufacturing Energy and Carbon Footprint

The CaCO_3_ cement manufacturing process (Figure 1) requires both electrical energy for mechanical processes (pre-processing, solubilization, filtration, CO_2_ capture, CaCO_3_ precipitation, dewatering, and aqueous recycling) and thermal energy for drying at approximately 130 °C. Based on pilot plant data, production of CaCO_3_ cement is calculated to require 2.8 to 3.9 MJ/kg when considering a range of dry and wet raw materials. This compares to the 4.6 to 5.6 MJ/kg energy consumed during Portland cement production, mainly from the calcination of limestone at 900 °C and the sintering process at 1450 °C [38]. Since producing CaCO_3_ cement from waste feedstocks does not require high temperature processes and can capture and store CO_2_ from industrial emissions, CaCO_3_ cement has a significantly lower CO_2_ footprint compared to Portland cement. The energy and carbon footprint for CaCO_3_ cement manufacturing will vary for different sites and plant scales due to factors such as electricity mix, fuel sources, and operational efficiency.

**Figure 9:**
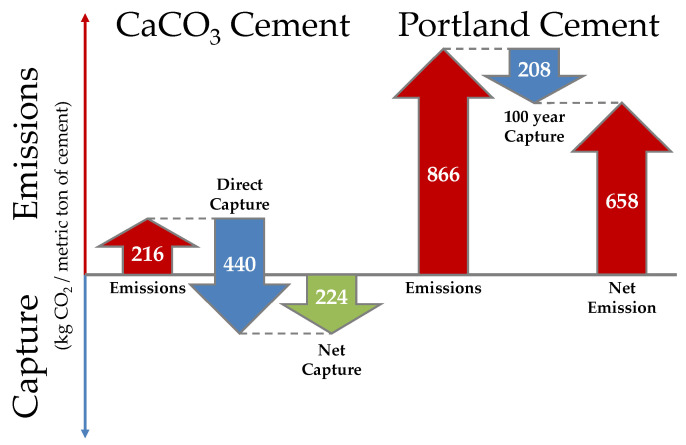
Carbon footprint of the pilot-scale CaCO_3_ cement manufacturing process compared to Portland cement manufacturing process (based on the upper range of energy consumption for the CaCO_3_ cement and average Portland cement emissions assuming 60% of emissions are from the calcination of limestone and 40% of those emissions are recaptured over 100 years [41,42,43]).

For the highest energy consumption case (3.9 MJ/kg CaCO_3_ cement), the electrical energy consumption is 92 kWh/metric ton of CaCO_3_ cement, and the natural gas consumption is 3.57 GJ/metric ton of CaCO_3_ cement. The plant operations that consume the most electricity are carbide lime sludge dissolution and filtration (39%) and CO_2_ absorption including flue gas compression (38%). 88% of the natural gas is consumed in the drying of the feedstock and product. Using a 2019 California grid carbon intensity of 175 kg CO_2_/MWh, the electricity results in emissions of 16 kg CO_2_/metric ton of CaCO_3_ cement. With an emission factor of 56 kg CO_2_/GJ, the natural gas combustion produces 200 kg CO_2_/metric ton of CaCO_3_ cement [39,40]. The total emissions are 216 kg CO_2_/metric ton of CaCO_3_ cement. Figure 9 compares the carbon captured and emitted during CaCO_3_ cement production to the CO_2_ emitted during Portland cement production. Since every metric ton of CaCO_3_ cement produced captures 440 kg of CO_2_ and manufacturing CaCO_3_ cement releases 216 kg of CO_2_, CaCO_3_ cement has a net capture of 224 kg of CO_2_/metric ton of CaCO_3_ cement produced. Substituting CaCO_3_ cement for Portland cement, results in a reduction of 882 kg of CO_2_/metric ton of cement utilized due to net emissions of 658 kg of CO_2_ avoided from Portland cement and a net capture of 224 kg of CO_2_ during the manufacture of CaCO_3_ cement when utilizing a waste Ca feedstock.

If curing is conducted at elevated temperature, additional energy input is required to heat the cementitious mixture to the curing temperature. For example, 1 m^3^ of concrete consisting of 305 kg CaCO_3_ cement, 153 kg water, and 1978 kg of silicious aggregate requires 147 MJ (0.48 MJ/kg CaCO_3_ cement) to heat from 20 to 80 °C. End of life uses for CaCO_3_ cement composites would be similar to those of Portland cement composites with the dominant recycling scheme being crushed into aggregate.

## 5. Conclusions

We have described a method of converting anthropogenic CO_2_ emissions into CaCO_3_ cement at a pilot scale. The CaCO_3_ cement gains approximately 40 MPa of strength during its conversion from vaterite to aragonite, which is 3× higher than previously reported results. The cement paste hardens and gains strength through the formation of a network of interlocking aragonite needles. The conversion and strength gain can be accelerated by elevated curing temperatures, e.g., full conversion and strength in 3 days at 80 °C. The CaCO_3_ cement system is particularly well-suited in applications that can take advantage of the inherent porosity, low pH, or rapid kinetics. The new cement system described is an environmentally sustainable alternative cement that can be carbon negative, meaning more CO_2_ can be captured during its manufacture than is emitted when utilizing a waste Ca feedstock.

## Figures and Tables

**Table 1:** Chemical composition of the oven-dried carbide lime sludge.

Oxide	Mass (%)
CaO	70.0
SiO_2_	2.4
Al_2_O_3_	1.7
MgO	0.1
Fe_2_O_3_	0.3
SO_3_	0.7
MnO	0
TiO_2_	0
P_2_O_5_	0
Cl	0
LOI ^1^	24.4

^1^ LOI is mass loss on ignition to 950 °C.

**Table 2:** Chemical (XRF) and mineral (QXRD) composition of the CaCO_3_ cement.

Oxide	Weight (%)	Mineral	Weight (%)
CaO	55.1	Vaterite	99.5
SiO_2_	0	Calcite	0.5
Al_2_O_3_	0		
MgO	0.1		
Fe_2_O_3_	0		
SO_3_	0.4		
MnO	0		
TiO_2_	0		
P_2_O_5_	0		
Cl	0.2		
LOI ^1^	44.1		

^1^ LOI is mass loss on ignition to 950 °C.

## Data Availability

All research data are available and can be furnished upon request.
